# Supplementary material for: Patterns of Intron Gain and Loss in Fungi
Source: PLoS Biol. 2004 Nov 30;2(12):e422. doi: 10.1371/journal.pbio.0020422 (PMC532390; doi:10.1371/journal.pbio.0020422)
Supplement: Table S1 — Also available at http://genes.mit.edu/NielsenEtAl/. (4.3 MB ZIP). [file pbio.0020422.st001.zip › NielsenEtAl/html/278.html]

AN1563.1.NCU04857.1.MG05506.1.FG02203.1


```
 CLUSTAL W (1.82) Multiple Sequence Alignments - Introns Inserted


Sequence 1: NCU04857.1	411 aa
Sequence 2: FG02203.1	370 aa
Sequence 3: MG05506.1	384 aa
Sequence 4: AN1563.1	378 aa
Alignment Length: 422 aa
Number Identitical Residues: 99 aa
Alignment Score (without introns) 5883


MG05506.1 	MTIRLARDEEPAAAVGQGLKGHKLLMLVPAPYMPPPEHLDYLRRRFPGLEIFAHARE~WA
NCU04857.1	MTLIKFSSLDNETKTNPTLKGHKLVFITPWE--PTPDYIDSLQKQFPDLVIECHKSE~WR
FG02203.1 	MTLIT-------TRTNKTLKGHKAVILLNTE--SPRTQVDVIQKRFPSLVIKTFIEK~KR
AN1563.1  	------------MGDAAVQKPHILVVIHRAF---PGFMTGEIRNEFPDAEVRFVTPQ0FL
          	                   * *  :.:       .    . ::..**.  :     :   

MG05506.1 	DRN-APAASADGGW--RWEDITILITG----SALPSKEEAVK~LQYVQLLSAGANHILQH
NCU04857.1	APGTGAGESPFPIE--QWKDVTIALT----FTYLPQPKDAPK~LQYVQLISAGANHILDH
FG02203.1 	GE---PLEPEFDEE--EWKDTTIILTAVATVHLLPRPEQAPH~LQYVQLTSAGADYFIKN
AN1563.1  	TRTDRFYFSAIGLTGPSWEDIVKQSTIIVTGRSLPNPEDAKH2IKFIHFFSAGLDKVIHD
          	        .     .. *:* .   *   :   **  ::* : :::::: *** : .:..

MG05506.1 	PLFAETDITFCTANGVHG~PQISEWVVGTLL~SFEHHL1PPLWDQQKES~RWEKKDAYQQ
NCU04857.1	PLFKETEVEFCTANGVHG2PQISEWLLLTYL~AFNHHL~PHYLALQQQA~HWSRAKSDS-
FG02203.1 	PLFTDTTIPICTASGVHG2LLTTQQGLKSPN1LDVGHD~DNLLEASTNS1QWHPLNTDPE
AN1563.1  	PVLTDSEIPVTTSSGIHG~PPIAEWTVMNWL~VASREY~SITYENQKKH~IWGSVDLYSH
          	*:: :: : . *:.*:**    ::  : .       .        . :   *   .    

MG05506.1 	-VTDSVGRRL2GVLGYGSIGRQT~ARLCKALGMDVHAYTLHPRHTAESRRDTSYAPPGTG
NCU04857.1	-IQDATAKTV~GILGYGAIGRQT~ARLAVAMGMRVHAFTLHPRPTPESRKDRSWAPSGMG
FG02203.1 	DATDTAGKRI2GILGYGSIGRQT~ARVSKALGLDIHAYTLHPRNTPESRRDNSYSPPGLG
AN1563.1  	GIQDHVGKKV~GILGYGSIGRQI1ARVAVSLGLSVYAYTASPKPTPESRRDRHYIIPGTG
          	.  * ..: : *:****:****  **:. ::*: ::*:*  *: *.***:*  :  .* *

MG05506.1 	DPEGLFPSKWFSGGSKQDLHRFLSSGLDVLVISTPLTDKTTHLLGPEEFEILSQR-----
NCU04857.1	DPDGIFPSKWFSGSSKEDLHSFLRSGLDLLVVSTPLTPGTKHLLGKEEFEVLYKASPTVK
FG02203.1 	DPEGEFPSKWFSGGSREDVHAFLDSGLDIVVIALPLTAKTQGLISKPELEILAKK-----
AN1563.1  	DADGTLPVSWHHGTSKASLHEFLSLGLDHIVVSLPLTPSTTHLLGAQEFAILAAN-----
          	*.:* :* .*. * *: .:* **  *** :*:: ***  *  *:.  *: :*        

MG05506.1 	-------------GTFVSNIARGPIIDTDALIDALNTGKIRGAALDVTDPEPLPKDHALW
NCU04857.1	VMNEETGKVEERGRTFVSNIARGPVVDTDELIEALEKGWIRGAALDVTDPEPLPDGHRLW
FG02203.1 	-------------RTFLINIARGPIVNTDDLIQALNSEKIVGAALDVTDPEPLPKKHPLW
AN1563.1  	-------KNPKHRNPYLTNISRGKVIDQDALIASLKSGELSGAALDVTDPEPLPEDHELW
          	       .  .   .:: **:** ::: * ** :*:.  : *************. * **

MG05506.1 	KAKNVTITPHLSSASNSYGKRIMGILEGNLEKLSQGKP-LMNR0DPEK~KTLHVTAGSAV
NCU04857.1	STRNVIVTPHVSGASTRYNERVLAILEENLGRVGRGER-LVNG0AKRE~RERSVFVSPSS
FG02203.1 	SAKNVTITPHISAISSFYFKRLFDIFGLNLERLGVGQTDLINK~VNR-~REGY-------
AN1563.1  	DTPNVQISPHVSSLGQEYFVRSFDIVRENLERVKDGLP-LINE~YKRE1QQQNGEA----
          	.: ** ::**:*. .  *  * : *.  ** ::  *   *:*    .. :          

MG05506.1 	G-----------
NCU04857.1	GTYLRASAQILI
FG02203.1 	------------
AN1563.1  	------------
          	
```
